# *In vivo* Antihypertensive and Antihyperlipidemic Effects of the Crude Extracts and Fractions of *Moringa stenopetala* (Baker f.) Cufod. Leaves in Rats

**DOI:** 10.3389/fphar.2016.00097

**Published:** 2016-04-21

**Authors:** Bekesho Geleta, Eyasu Makonnen, Asfaw Debella, Ashenif Tadele

**Affiliations:** ^1^Directorate of Traditional and Modern Medicine Research, Ethiopian Public Health InstituteAddis Ababa, Ethiopia; ^2^Department of Pharmacology, School of Medicine, College of Health Sciences, Addis Ababa UniversityAddis Ababa, Ethiopia

**Keywords:** antihypertensive, fructose, *in vivo*, *Moringa stenopetala*, antihyperlipidemic

## Abstract

**Background:** *Moringa stenopetala* (Baker f.) Cufod. is a medicinal plant that has been used in Ethiopian traditional medicine as a remedy for treatment of hypertension and diabetes. The aim of this study was to evaluate antihypertensive and antihyperlipidemic effect in fructose induced hypertensive rats.

**Methods:** Rats were randomly divided into control and treatment groups (*n* = 6). Treatment groups were given daily extracts (250, 500, and 1000 mg/kg) orally with fructose. Whereas, positive, negative and normal control groups were received captopril (20 mg/kg/day with fructose), only fructose (66% w/v *ad libitum*) and distilled water *ad libitum* for 15 days, respectively. The blood pressure was measured every 5th day using tail cuff blood pressure analyzer, and on the 16th day the blood was sampled to evaluate antihyperlipidemic effect using clinical chemistry analyzer.

**Results:** The study showed that aqueous and 70% ethanol extracts significantly prevented blood pressure increment in a dose dependent manner comparable to that of the standard drug. Similarly, the extracts suppressed increment in lipid profile (cholesterol, glucose, and triglycerides) compared with negative control. The biochemical test revealed that extracts produced a rise in liver but no effect on kidney function indicators compared with normal control.

**Conclusion:** These findings revealed that both crude extracts of *M. stenopetala* (Baker f.) Cufod. possess antihypertensive and antihyperlipidemic effect.

## Introduction

Hypertension is a leading cause of CVDs such as myocardial infarction and stroke worldwide. The proportion of the global burden of disease attributable to hypertension has significantly increased from about 4.5% (nearly1 billion adults) in 2000, to 7% in 2010 (WHO, 2008; AU, 2013). Hypertension leads to complications with considerable morbidity and mortality which is responsible for at least 45% of deaths due to heart disease and 51% of deaths due to stroke (WHO, 2008). Hypertension accounts for 9.4 million deaths worldwide every year. At the beginning of the twentieth century, CVD was responsible for less than 10% of all deaths worldwide, but by 2008, the figure had risen to 30%. The number of people with the condition rose from 600 million in 1980 to 1 billion in 2008 (WHO, 2011). Moreover, the number of people with uncontrolled hypertension has increased to around 1 billion worldwide in the past three decades (Danaei et al., 2011). About 80% of the global burden of CV death occurs in low and middle income countries. This is nearly as many deaths as caused by HIV, malaria, and TB (Gaziano et al., 2006; Lopez et al., 2006). This makes hypertension the single most important cause of morbidity and mortality globally and highlights the urgent need of action to address the problem (AU, 2013). Hypertension was almost non-existent in African adult societies in the first half of the twentieth century, the prevalence increased significantly over the past two to three decades to more than 40% (about 80 million) and projections based on current epidemiological data suggest that this figure will rise to 150 million by 2025 (AU, 2013). WHO projects that over the next 10 years Africa will experience the largest increase in death rates from CVD and therefore the negative economic impact of CVD will be more felt on the continent (Alwan, 2011).

**Figure 1:**
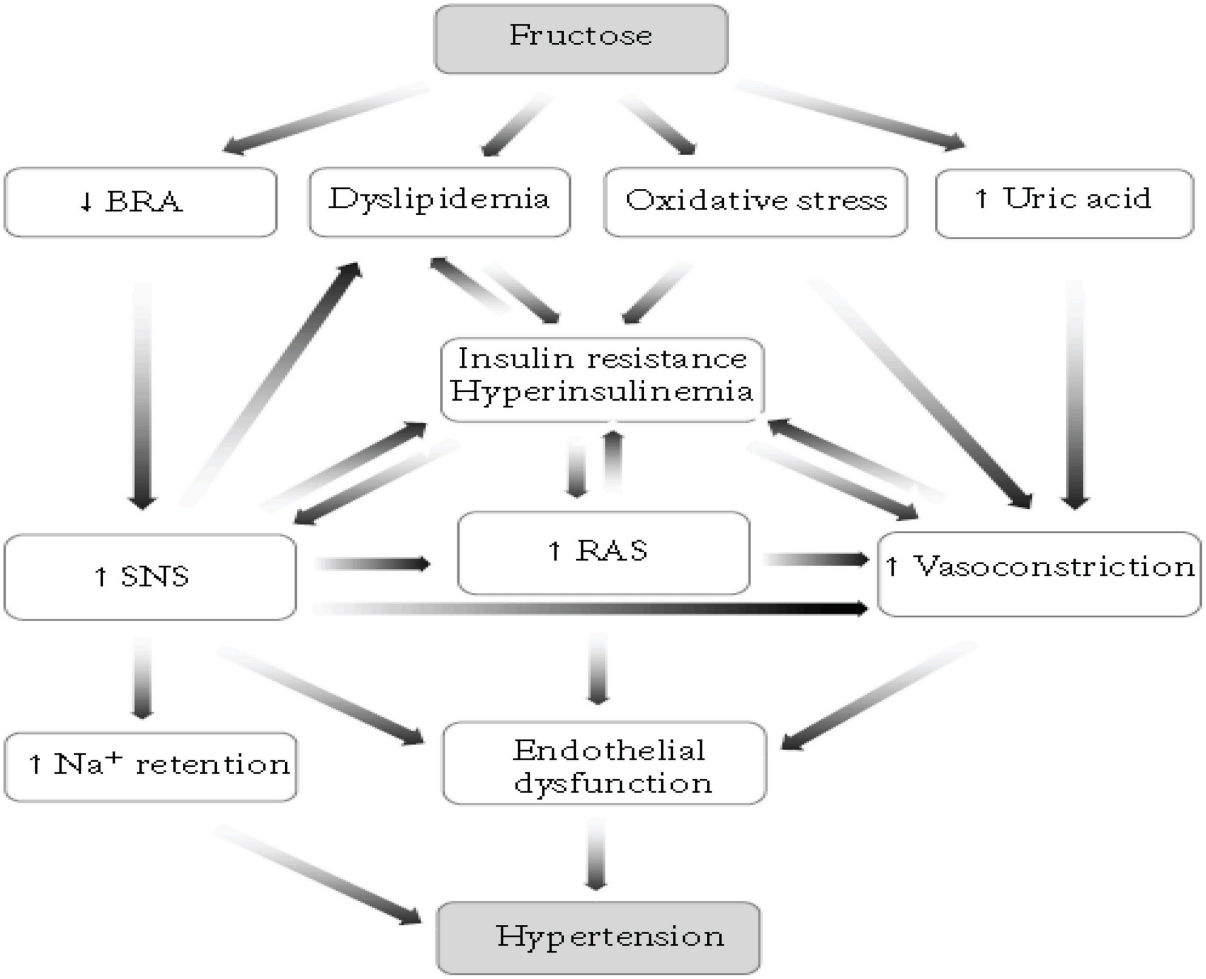
**The proposed mechanisms by which fructose feeding results in hypertension**.

There are different models of inducing hypertension in animals. Such as, renovascular hypertension, dietary hypertension, endocrine hypertension, neurogenic hypertension, psychogenic hypertension, genetic hypertension, and other models (Kaur et al., 2011). Among these, the dietary induction of hypertension is the most commonly employed in rodents and the mechanism behind is described below (Figure 1; Abdulla et al., 2011). Increases in dietary carbohydrate intake such as fructose, sucrose, or glucose can raise BP in normal rats (Hwang et al., 1987). High fructose consumption will produce a model of the metabolic syndrome with hypertension, insulin resistance, hyperinsulinemia, hyperlipidemia, and hypertriglyceridemia in normal rats and this greatly accelerates progression of chronic kidney disease (Gersch et al., 2007).

Clinically, various antihypertensive drugs such as ACEIs, ARBs, diuretics, CCBs, β blockers, alpha-1 blockers, central α-2 agonists, non-selective α and β blockers, and direct vasodilators have been used to manage hypertension and to alleviate symptoms (Benowitz, 2012). ACEIs and ARBs are ideal first line antihypertensive agents in individuals with type 2 diabetes (Tashko and Gabbay, 2010). Despite the availability of a wide range of antihypertensive drugs, hypertension and its complications are still important causes of morbidity and mortality in Africa. More than 50% of treated hypertensive patients have a BP level greater than 140/90 mm Hg (uncontrolled hypertension; Salako et al., 2003). Moreover, the efficacy of these drugs are only 40–60%, and usually two or more antihypertensive drugs from different categories need becombined to achieve optimal results, however side effects from these medications are important concerns (Du and Chen, 2005).

Various herbal preparations have been claimed to have benefit for hypertension. *Moringa stenopetala* (Baker f.) Cufod. is one of those plants used in Ethiopia. It grows abundantly in south western Ethiopia at an altitude range of 1000–1800 m where the leaves are eaten as vegetable besides its medicinal use (Arora et al., 2013). The species is known by different vernacular names such as “Shiferaw” in Amharic, “Aleko” in Gamugna, and “Cabbage tree” in English (Mekonnen and Gessesse, 1998). It has been reported that, *M. stenopetala* (Baker f.) Cufod. has hypotensive (Mengistu et al., 2012), antihyperglycemic (Toma et al., 2012, 2015; Sileshi et al., 2014) and also has a nutritional value (Abuye et al., 2004). The objective of the present study is, therefore, to investigate the antihypertensive and antihyperlipidemic effects of extracts and fractions of *M. stenopetala* (Baker f.) Cufod. leaves in fructose induced hypertensive rats.

## Materials and methods

### Drugs and chemicals

Ethyl Acetate (lot no: 8114/4, Park Scientific Limited, Northampton, UK), Absolute Ethanol (lot no: E35070/2, WINLAB, UK), Lead Acetate (lot no: V9H4049, Celtic Chemicals, South Wales, UK), Ammonia Solution (lot no: 9457, Scientific limited, UK), Dinitro-2-4- Phenylhydrazine (lot no: 231523, VWR Prolabo chemicals, USA), Sulfuric Acid (lot no: 8114/1, Scientific limited, UK), Chloroform (lot no: 8114/1, Scientific limited, UK), Hydrochloric Acid (lot no: 2571, Parchem fine and specialty chemicals, UK), D-Fructose (lot no: SL54161301, LobaChemie, India), Captopril (lot no: 48794, EPSITRON Limited, Nicosia, Cyprus) were used in the study. All the drugs, chemicals, and reagents used complied with the required standard and were of analytical grade.

### Instruments and apparatus

Lyophilizer/ Freeze dry system (Labconco, 12 L Console Freeze Dry 230v-60 (7754040), Freeze Dry System, USA), BP analyzer (Model 179, USA), Centrifuge (Rotant 98, Hettich, Zentrifugen, UK), Clinical chemistry analyzer (Cobas-e-411, HITACHI, ROCHE, Germany).

### Plant material

The fresh *M. stenopetala* (Baker f.) Cufod. leaves were collected from Southern Ethiopia around Arbaminch, about 500 km far from Addis Ababa on September 2014. The plant material was authenticated by a taxonomist in the EPHI and a voucher number AL-001 was deposited in the herbarium for future reference.

### Experimental animals

A statement of ethics approval is obtained from Scientific and Ethical Review Committee of EPHI. The experiments were performed on adult, healthy male Wistar rats (*Rattus norvegicus)* weighing 150–200 g bred and obtained from the EPHI. All the animals used for this study were kept in standard animal cages and maintained under laboratory conditions of temperature (22 ± 3°C), relative humidity (40–70%) and 12 h day-12 h night and had free access to food (standard pellet diet) and water *ad libitum.* The animals were treated humanely throughout the study period and were kept in a well-controlled area according to the guideline for use and care of animals (National Research Council, 2011).

### Plant material preparation and extraction

Fresh *M. stenopetala* leaves were garbled, chopped, dried under shade (at room temperature), grinded to powder using mortar and pestle and stored in cool and dry place. Weighed amounts of 1.208 and 2.130 Kg powdered leaves were kept in Erlenmeyer flasks and macerated with water (distilled and deionized) and 70% ethanol at room temperature under a rotator shaker until exhaustion for 4 and 72 h, respectively. The 70% EtOH extract was filtered using cotton gauze and then with Whatman filter paper No.1. The filtrate was concentrated under reduced pressure using Rota vapor. The semidried residue was kept on a water bath at 40°C overnight and then with a Lyophilizer to completely remove the solvent residue. The AQ crude extract was filtered using a Whatman filter paper No.1, kept in refrigerator overnight to freeze and lyophilized to remove the water. The total yield of the AQ and 70% EtOH extract were calculated 17.1 and 4.9% (w/w), respectively.

About 178.95 g of dried AQ crude extract were defatted by petroleum ether and partitioned with EtAc, the solvent was removed using Rota vapor and Lyophilizer to obtain EtAc (18.6% w/w yield) and AQ residue (35% w/w yield), respectively. The dried extracts were kept in a refrigerator until used for the experiment.

### Phytochemical screening

All extracts used for the *in vivo* study were subjected to phytochemical screening following methods described by Tiwari et al. (2011). The extracts along with negative controls were tested for the presence of alkaloids, saponins, polyphenols, flavonoids, coumarins, terpenoids, anthraquinones, tannins, phytosterols, and glycosides as follows:

- Alkaloids
  One and half milliliter of 10% HCl was added to 0.5 mg of the extracts in a test tube. The mixture was heated for 20 min. It was then cooled and filtered. To 1 ml of the filtrate five drops Mayers and Draggendorff's reagents each were added. Formation of cream and orange colored precipitates respectively indicates the presence of alkaloids in the extracts.
- Saponins
  Froth test: An aqueous solution of 0.5 mg of the extract in a test tube was vigorously shaken for 2 min. Foam which persisted for 30 min and doesn't disappear upon warming was taken as an indication of the presence of saponin in the extract.
- Polyphenols (Phenolic compounds)
  Three drops of a mixture of 1 ml 1% FeCl3 and 1% K_3_Fe(CN)_6_ each were added to 2 ml of extracts. Formation of green or blue color was taken as an indication of the presence of polyphenols.
- Flavonoids
  To 2 ml of aqueous solution of the extract four drops of 2% lead acetate solution was added. Development of yellow or orange color confirms the presence of flavonoids.
- Coumarins
  Two milliliter of 10% ammonia solution was added to 5 ml concentrated alcoholic solution of the extracts. The occurrence of an intensive fluorescence under UV light indicates the presence of coumarin derivatives.
- Terpenoids (Ketonic)
  One milliliter of 2, 4-dinitrophenylhydrazine solutions (0.5 g dissolved in 100 ml of 2 M HCl) was added to 2 ml aqueous solution of the extract. Formation of yellow-orange coloration indicates the presence of a ketonic terpenoids.
- Anthraquinones
  Borntrager's test: Five milliliter of the extract was dried and shaken with 3 ml petroleum ether. The filtrate was added to 2 ml of a 25% ammonia solution. The mixture was shaken and formation of a red coloration was taken as an indication of the presence of free anthraquinones.
- Tannins
  Three drops of 5% ferric chloride solution was added to 1 ml of the extract solution in water. A greenish or blue coloration or precipitation was taken as indication of the presence of tannins.
- Phytosterols and Withanoids
  Five drops of 3% vanillin in conc. H_2_SO4 was added to a concentrated chloroform solution of extracts. Formation of a rose or reddish brown color indicates the presence of anoids or phytosterols.
- Test for Glycosides (Keller-Killiani Test)
  To 0.5 g of each extract suspended in 5 ml water, 2 ml of glacial acetic acid containing one drop of ferric chloride hexahydrate (FeCl_3_.6H_2_O) solution was added. This was mixed with 1 ml of concentrated sulfuric acid and observed for a brown ring at the interface or a violet ring below the brown ring; alternatively acetic acid was added and observed for a greenish ring above the brown ring which gradually spread throughout this layer.

### Evaluation of in *vivo* antihypertensive effect

Among different models available, for the present study, the dietary induction of hypertension in male Wistar rats was employed using 66% w/v D-Fructose according to methods described by Jena et al. (2013).

Each rat wastrained and acclimatized to the restrainer and transducer, for about 15 min each day before the experiment. The rat was restrained in a low-stress environment and allowed to enter the holder freely at least 10–15 min prior to obtaining BP measurements. The animal's nose was made to protrude through the front of the nose cone allowing for comfortable breathing and the tail of the animal was fully extended to exit through the rear hatch opening of the holder. The rat was warmed but not heated using restrainer, the room temperature was maintained about 32–35.4°C, reduce stress and the blood flow to the tail was enhanced to acquire a BP signal. The rat never had its head bent sideways or its body compressed against the back hatch. The animal's temperature was monitored throughout the experiment (Malkoff, 2005).

Male Wistar rats were randomly divided into groups with six animals (*n* = 6). Normal control rats (Group 1) received distilled water *ad libitum* only, negative control rats (Group 2) received 66% w/v D-Fructose *ad libitum* only, positive control rats (Group 3) received captopril (20 mg/kg/day) with 66% w/v D-Fructose *ad libitum* and treatment rats, Group 4 received 250 mg/kg AQ crude extract, Group 5 received 500 mg/kg AQ crude extract, Group 6 received 1000 mg/kg AQ crude extract, Group 7 received 250 mg/kg 70%EtOH crude extract, Group 8 received 500 mg/kg 70%EtOH crude extract, Group 9 received 1000 mg/kg 70%EtOH crude extract, Group 10 received 250 mg/kg AQ residue of AQ crude extract, Group 11 received 500 mg/kg AQ residue of AQ crude extract, Group 12 received 1000 mg/kg AQ residue of AQ crude extract, Group 13 received 250 mg/kg EtAc fraction of AQ crude extract, Group 14 received 500 mg/kg EtAc fraction of AQ crude extract, Group 15 received 100 mg/kg EtAc fraction of AQ crude extract with 66% w/v D-Fructose *ad libitum* for 15 days. The dose was selected based on the acute toxicity study; LD_50_ was greater than 5000 mg/kg (Geleta et al., 2016). The extracts were prepared for administration by weighing a required amount of dried extracts and dissolving in a suitable vehicle (distilled water). Then, the uniformly dissolved volume of extract was administered to rats using oral gavage.

SBP and MAP were measured on the 1st day of experiment before being induced using (66% w/v) D-Fructose, and was stated as a BP_0_. Rats with SBP_0_ ≤ 120 mmHg, MAP_0_ ≤ 100 mmHg and DBP_0_ ≤ 91 mmHg were considered normotensive and were given (66% w/v) D-Fructose *ad libitum* except those served as normal control. Then BP was measured every 5th days to have D_5_, D_10_ and D_15_ BP readings using BP analyzer. The SBP and MAP were read from the pulse tracings and DBP was calculated using formula –1.

$$\begin{array}{l} \text{DBP}=\left(3\text{MBP}-\text{SBP}\right)∕2\ldots \ldots \ldots \ldots \ldots \ldots .1 \end{array}$$

Every measurement was taken in triplicate and the average value was reported.

On the 16th day, the blood was collected in vacutainer tube by cardiac puncture from night fasted cervical dislocated rats. The serum was separated after centrifugation at 3000 rpm for 10 min. The serum lipid profile (TC, BG, TG) were assayed using methods described by the manufacturer (Roche diagnostics, Germany) using COBA-e-411 Clinical chemistry analyzer instrument.

### Statistical analysis

All experimental data's were expressed as mean values (measurement of BP or % relaxation) ± S.E.M and were subjected to biostatistical interpretation by SPSS windows version 20 statistical packages all the way through a one-way ANOVA followed by *post-hoc* test (Tukey Test) for multiple comparisons of the mean differences and responses of different drugs and extracts. Statistical significance of *P* < 0.05 were considered as level of significance.

## Results

### Phytochemical screening

Basic investigations of the extracts for their major phytocompounds is vital as the active principles of many drugs are these secondary metabolites found in plants. The various phytochemical screening tests performed on the crude extracts and solvent fractions *M. stenopetala* leaves revealed the presence of different secondary metabolites (Table 1).

**Table 1:** **Phytochemical screening of crude extracts and solvent partitions of *M. stenopetala* (Baker f.) Cufod. Leaves**.

**Type of extract**	**Alkaloids**	**Saponins**	**Polyphenols**	**Flavonoids**	**Coumarins**	**Terpenoids**	**Anthraquinones**	**Tannins**	**Phytosterols**	**Cardiac glycosides**
AQ	+	+	+	+	+	+	+	+	+	+
AQ partition residue of AQ	−	+	−	+	+	+	+	+	+	−
EtAc partition of AQ	−	+	+	−	−	+	−	+	+	−
70% EtOH	+	−	+	+	+	+	+	+	+	+
Negative control (vehicle)	−	−	−	−	−	−	−	−	−	−

### In *vivo* antihypertensive and antihyperlipidemic activity

#### Effect on blood pressure

The negative control showed significant increase in SBP, MAP, and DBP compared with normal control (*P* < 0.001) and positive control (*P* < 0.001) in the D_5_, D_10,_ and D_15_ of the experiment (Tables 2–**4**). Those groups that received daily oral administration of 1000 mg/kg of AQ crude, 70% EtOH crude and AQ residue of AQ extract prevented a rise in SBP and did not show significant difference in the D_5_ of the experiment. In the D_10_ of experiment, 1000 mg/kg/day oral administration of the crude extracts prevented a rise in SBP, did not show significant difference in SBP compared with normal and positive control. After consecutive oral daily administration for 15 days, all treatment groups showed significant increase in SBP compared with normal control (*P* < 0.01) and positive control (*P* < 0.05; Table 2).

**Table 2:** **Effect of crude extracts and solvent fractions of ***M. stenopetala*** (Baker f.) Cufod. leaves on SBP in D-Fructose (66% w/v ***ad libitum***) induced rats**.

**Substance administered**	**Dose (mg/kg)**	**SBP**			
		**D_1_ (mmHg)**	**D_5_ (mmHg)**	**D_10_ (mmHg)**	**D_15_ (mmHg)**
D-Fructose	66% w/v *ad libitum*	113.50 ± 1.52	132.67 ± 0.71^***^^c^^,^^*^^c^	144.33 ± 1.33^***^^c^^,^^*^^c^	163.67 ± 1.12^***^^c^^,^^*^^c^
Captopril	20	113.83 ± 2.12	124.83 ± 1.19^**^^c^^,^^*^^c^	120.67 ± 0.88^**^^c^^,^^*^^a^	120.50 ±0.76^**^^c^
AQ crude	250	114.17 ± 1.40	129.50 ± 1.38^*^^c^	138.67 ± 0.88^**^^c^^,^^***^^c^^,^^*^^c^	149.67 ± 1.28^**^^c^^,^^***^^c^^,^^*^^c^
	500	112.50 ± 1.20	126.83 ± 0.95^**^^b^^,^^*^^c^	132.17 ± 0.70^**^^c^^,^^***^^c^^,^^*^^c^	136.50 ± 0.76^**^^c^^,^^***^^c^^,^^*^^c^
	1000	116.83 ± 1.78	121.83 ± 0.60^**^^c^	123.00 ± 0.58^**^^c^	124.67 ± 0.88^**^^c^^,^^***^^a^^,^^*^^b^
70% EtOH crude	250	116.17 ±1.01	138.67 ± 0.88^**^^b^^,^^***^^c^^,^^*^^c^	146.83 ± 0.60^***^^c^, ^*^^c^	157.17 ± 0.95^**^^c^^,^^***^^c^^,^^*^^c^
	500	114.83 ± 1.96	130.50 ± 0.76^***^^a^^,^^*^^c^	137.50 ± 0.76^**^^c^^,^^***^^c^^,^^*^^c^	142.50 ± 0.76^**^^c^^,^^***^^c^^,^^*^^c^
	1000	115.17 ± 1.97	119.00 ± 1.39^**^^c^^,^^***^^b^	124.33 ± 0.49^**^^c^^,^^*^^c^	133.50 ± 0.76^**^^c^^,^^***^^c^^,^^*^^c^
EtAc fraction of AQ crude	250	118.33 ± 0.88	141.33 ± 0.80^**^^c^^,^^***^^c^^,^^*^^c^	151.17 ± 0.60^**^^c^^,^^***^^c^^,^^*^^c^	161.17 ± 0.60^***^^c^^,^^*^^c^
	500	119.67 ± 0.88	134.17 ± 0.70^***^^c^^,^^*^^c^	141.17 ±0.483^***^^c^^,^^*^^c^	151.50 ± 0.76^**^^c^^,^^***^^c^^,^^*^^c^
	1000	116.50 ±1.48	124.33 ± 1.33^**^^c^^,^^*^^a^	134.50 ± 0.76^**^^c^^,^^***^^c^^,^^*^^c^	145.33 ± 0.88^**^^c^^,^^***^^c^^,^^*^^c^
AQ residue of AQ crude	250	116.83 ± 1.78	137.83 ± 0.60^**^^a^^,^^***^^c^^,^^*^^c^	142.17 ± 0.70^***^^c^^,^^*^^c^	156.00 ± 0.58^**^^c^^,^^***^^c^^,^^*^^c^
	500	116.83 ± 1.54	132.67 ± 0.88^***^^c^^,^^*^^c^	139.50 ± 0.42^**^^b^^,^^***^^c^^,^^*^^c^	146.17 ± 0.60^**^^c^^,^^***^^c^^,^^*^^c^
	1000	117.33 ± 1.63	124.17 ± 1.01^**^^c^	131.17 ± 0.60^**^^c^^,^^***^^c^^,^^*^^c^	139.50 ± 0.43^**^^c^^,^^***^^c^^,^^*^^c^

Results are expressed as mean ± SEM (n = 6),

* compared with normal control (baseline or basal value);

** compared with negative control;

*** compared with positive control.

a P < 0.05.

b P < 0.01.

c P < 0.001.

Groups that received, 1000 mg/kg/day oral administration of all extracts except the one that received AQ extract showed significant increase (*P* < 0.001) in MAP compared with normal control in the D_5_ of the experiment. In the D_10_ of experiment, daily oral administration of 1000 mg/kg of crude extracts prevented a rise in MAP in a similar manner with positive control. After consecutive oral daily administration for 15 days, group that received 1000 mg/kg of AQ crude extract didn't show significant difference in MAP compared with normal control as well as positive control (Table 3).

**Table 3:** **Effect of crude extracts and solvent fractions of *M. stenopetala* (Baker f.) Cufod. leaves on MAP in D-Fructose (66% w/v *ad libitum*) induced rats**.

**Substance administered**	**Dose (mg/kg)**	**MAP**			
		**D_1_ (mmHg)**	**D_5_ (mmHg)**	**D_10_ (mmHg)**	**D_15_ (mmHg)**
D-Fructose	66% w/v *ad libitum*	96.95 ± 1.00	108.02 ± 0.80^***^^c^^,^^*^^c^	118.45 ±0.68^***^^c^^,^^*^^c^	130.88 ±0.50^***^^c^^,^^*^^c^
Captopril	20	97.40 ± 0.81	100.83 ± 0.99^**^^c^	99.55 ±0.74^**^^c^	99.38 ± 065^**^^c^
AQ crude	250	97.07 ± 0.75	106.83 ± 0.99^***^^c^^,^^*^^c^	112.88 ± 0.76^**^^c^^,^^***^^c^^,^^*^^c^	121.35 ±0.74^**^^c^^,^^***^^c^^,^^*^^c^
	500	97.17 ± 0.59	103.15 ± 0.50^**^^c^^,^^*^^c^	105.05 ± 0.43^**^^c^^,^^***^^c^^,^^*^^c^	107.27 ± 0.84^**^^c^^,^^***^^c^^,^^*^^c^
	1000	99.38 ± 0.68	101.27 ±0.58^**^^c^	100.23 ± 0.37^**^^c^	98.57 ± 0.75^**^^c^
70% EtOH crude	250	98.27 ± 0.44	110.45 ±0.58^***^^c^^,^^*^^c^	117.73 ± 0.56, ^***^^c^^,^^*^^c^	125.40 ± 0.48^**^^c^^,^^***^^c^^,^^*^^c^
	500	97.28 ± 0.71	106.15 ± 0.48^***^^c^^,^^*^^c^	111.72 ± 0.63^**^^c^^,^^***^^c^^,^^*^^c^	113.62 ± 0.34^**^^c^^,^^***^^c^^,^^*^^c^
	1000	98.72 ± 0.79	100.68 ±0.81^**^^c^^,^^*^^c^	101.68 ± 0.53^**^^c^	106.18 ± 0.55^**^^c^^,^^***^^c^^,^^*^^c^
EtAc fraction of AQ crude	250	99.77 ±0.43	111.70 ± 0.37^**^^a^^,^^***^^c^^,^^*^^c^	119.15 ± 0.85^***^^c^^,^^*^^c^	128.15 ± 0.51^***^^c^^,^^*^^c^
	500	99.55 ± 0.75	108.17 ± 0.49^***^^c^^,^^*^^c^	114.50 ± 0.52^**^^b^^,^^***^^c^^,^^*^^c^	123.27 ± 0.32^**^^c^^,^^***^^c^^,^^*^^c^
	1000	99.15 ± 0.70	105.00 ± 0.76^***^^b^^,^^*^^c^	112.50 ± 0.61^**^^c^^,^^***^^c^^,^^*^^c^	119.67 ± 0.44^**^^c^^,^^***^^c^^,^^*^^c^
AQ residue of AQ crude	250	99.17 ± 1.64	109.62 ± 0.56^***^^c^^,^^*^^c^	115.83 ± 0.69^***^^c^^,^^*^^c^	125.33 ± 0.47^**^^c^^,^^***^^c^^,^^*^x^c^
	500	97.95 ±0.83	108.12 ± 0.57^*^^c^	113.27 ±0.48^**^^c^^,^^***^^c^^,^^*^^c^	120.28 ±0.42^**^^c^^,^^***^^c^^,^^*^^c^
	1000	98.80 ± 0.89	103.38 ±0.57^**^^b^^,^^*^^a^	108.03 ± 0.49^**^^c^^,^^***^^c^^,^^*^^c^	113.48 ±0.47^**^^c^^,^^***^^c^^,^^*^^c^

Results are expressed as mean ± SEM (n = 6),

* compared with normal control;

** compared with negative control;

*** compared with positive control.

a P < 0.05.

b P < 0.01.

c P < 0.001.

Daily oral administration of 500 and 1000 mg/kg/day of AQ crude, 70% EtOH crude, and AQ residue of AQ crude extracts prevented a rise in DBP compared with normal control in the D_5_ of the experiment. Groups that received AQ crude extract (500 and 1000 mg/kg/day) and 70% EtOH crude extract (1000 mg/kg/day) prevented a rise in DBP when compared with normal control in the D_10_ of the experiment. After consecutive oral daily administration for 15 days, groups that received 1000 mg/kg AQ crude extract showed significant decrease (*P* < 0.05) in DBP compared with normal control (Table 4).

**Table 4:** **Effect of crude extracts and solvent fractions of *M. stenopetala* (Baker f.) Cufod. leaves on DBP in D-Fructose (66% w/v *ad libitum*) induced rats**.

**Substance Administered**	**Dose (mg/kg)**	**DBP**			
		**D_1_ (mmHg)**	**D_5_ (mmHg)**	**D_10_ (mmHg)**	**D_15_ (mmHg)**
D-Fructose	66% w/v *ad libitum*	88.67 ±1.36	95.67 ± 0.88^***^^c^^,^^*^^c^	105.50 ± 0.76^***^^c^^,^^*^^c^	114.50 ± 0.76^***^^c^^,^^*^^c^
Captopril	20	89.17 ± 0.87	88.83 ± 1.01^**^^c^	89.00 ± 0.97^**^^c^	88.83 ±0.95^**^^c^
AQ crude	250	88.50 ±0.76	95.50 ± 0.99^***^^c^^,^^*^^c^	100.00 ± 0.97^**^^c^^,^^***^^c^^,^^*^^c^	107.17 ± 0.60^**^^c^^,^^***^^c^^,^^*^^c^
	500	89.50 ± 0.76	91.33 ± 0.67^**^^a^	91.50 ±0.76^**^^c^	92.67 ± 1.45^**^^c^^,^^***^^a^
	1000	90.67 ±0.67	91.00 ± 0.58^**^^b^	88.83 ±0.31^**^^c^	85.50 ± 0.76^**^^c^^,^^*^^a^
70% EtOH crude	250	89.33 ± 1.05	96.33 ± 0.88^***^^c^^,^^*^^c^	103.17 ± 0.60^***^^c^^,^^*^^c^	109.50 ± 0.43^**^^c^^,^^***^^c^^,^^*^^c^
	500	88.50 ± 0.76	94.00 ±0.58***^b^,*^b^	98.83 ± 0.60^**^^c^^,^^***^^c^^,^^*^^c^	99.17 ± 0.60^**^^c^^,^^***^^c^^,^^*^^c^
	1000	90.50 ±0.76	91.50 ± 0.76^**^^a^	90.33 ± 0.88^**^^c^	92.5 ±0.76^**^^c^
EtAc fraction of AQ crude	250	90.50 ±0.76	96.83 ± 0.60^***^^c^^,^^*^^c^	103.17 ± 1.08^***^^c^^,^^*^^c^	111.67 ± 0.67^***^^c^^,^^*^^c^
	500	89.50 ±0.76	95.17 ± 0.60^***^^c^^,^^*^^b^	101.17 ± 0.60^**^^a^^,^^***^^c^^,^^*^^c^	109.17 ± 0.60^**^^c^^,^^***^^c^^,^^*^^c^
	1000	90.50 ± 0.76	95.33 ± 0.88^***^^c^^,^^*^^a^	101.50 ± 0.76^**^^a^^,^^***^^c^^,^^*^^a^	106.83 ± 0.60^**^^c^^,^^***^^c^^,^^*^^c^
AQ residue of AQ crude	250	90.33 ± 1.80	95.50 ± 0.76^**^^c^^,^^***^^c^^,^^*^^b^	102.67 ± 0.88^***^^c^^,^^*^^c^	110.00 ± 0.58^**^^b^^,^^***^^c^^,^^*^^c^
	500	88.50 ± 0.76	95.83 ± 0.79^***^^c^^,^^*^^c^	100.17 ± 0.79^**^^b^^,^^***^^c^^,^^*^^c^	107.33 ± 0.49^**^^c^^,^^***^^c^^,^^*^^c^
	1000	89.50 ± 0.76	93.00 ± 0.82^***^^a^	96.50 ±0.76^**^^c^^,^^***^^c^^,^^*^^c^	100.50 ±0.76^**^^c^^,^^***^^c^^,^^*^^c^

Results are expressed as mean ± SEM (n = 6),

* compared to normal control;

** compared to negative control;

*** compared to positive control.

a P < 0.05.

b P < 0.01.

c P < 0.001.

#### Effect on total cholesterol, glucose, and triglyceride plasma levels

The negative control showed significant increase in serum TC (*P* < 0.01), BG (*P* < 0.001) and TG (*P* < 0.001) level compared with normal control. Although all extracts prevented a rise in TG level in a dose dependent manner, there was significant increase (*P* < 0.001) compared with normal control. However, groups that received 500 and 1000 mg/kg/day of all extracts showed significant difference (*P* < 0.001) in TG level compared with negative control. Groups that received AQ crude extract (500 and 1000 mg/kg/day), AQ residue of AQ crude extract (1000 mg/kg/day) and normal control showed significant difference (*P* < 0.05) in BG level compared with negative control (Table 5).

**Table 5:** **Effect of crude extracts and solvent fractions of *M. stenopetala* (Baker f.) Cufod. leaves on lipid profiles (TC, BG, and TG plasma level) in D-Fructose (66% w/v *ad libitum*) induced rats**.

**Substance administered**	**Dose (mg/kg)**	**Lipid Profiles**		
		**CHOL (mg/dl)**	**GLUC (mg/dl)**	**TG (mg/dl)**
D-Fructose	66%w/w *ad libitum*	67.37 ± 5.87^*^^b^	142.00 ± 4.49^*^^c^	333.97 ± 32.92^*^^a^^,^^*^^c^
Captopril	20	63.73 ± 5.47	121.98 ± 19.20	298.23 ± 28.62^*^^c^
AQ crude	250	62.68 ± 6.00	132.83 ± 3.77^*^^b^	310.30 ± 8.09^*^^c^
	500	59.17 ± 5.52	109.15 ± 3.45^**^^a^	297.33 ± 12.78^*^^c^
	1000	57.75 ± 4.11	94.28 ± 2.76^**^^c^	194.53 ± 20.50^**^^a^^,^^*^^a^
70% EtOH crude	250	61.50 ± 6.95	141.02 ± 2.17^*^^c^	329.43 ± 39.25^*^^c^
	500	58.28 ± 4.21	129.48 ± 9.39^*^^a^	297.58 ± 17.17^*^^c^
	1000	57.82 ± 2.13	114.67 ± 2.73	267.38 ± 13.37^*^^c^
EtAc fraction of AQ crude	250	56.72 ± 8.84	139.17 ± 2.21^*^^c^	252.70 ± 1.95^*^^c^
	500	58.22 ± 2.85	130.92 ± 4.36^*^^a^	258.55 ± 34.22^*^^c^
	1000	60.72 ± 2.06	119.80 ± 3.34	317.22 ± 19.57^*^^c^
AQ fraction of AQ crude	250	57.05 ± 4.81	132.53 ± 1.75^*^^b^	215.40 ± 40.34^*^^b^
	500	54.05 ± 3.93	122.15 ± 4.26	310.27 ± 20.80^*^^c^
	1000	59.57 ± 4.35	96.70 ± 4.26^**^^a^	298.44 ± 7.18^*^^c^
Negative Control	Distilled Water	51.20 ± 4.69^**^^b^	97.60 ± 2.89^**^^a^	56.50 ± 3.13^**^^c^^,^^***^^c^

*Results are expressed as mean ± SEM (n = 6)*,

* compared with normal control;

** compared with negative control;

*** compared to positive control.

a P < 0.05.

b P < 0.01.

c P < 0.001.

The percentage difference BG level between AQ crude extract and AQ residue of AQ extract (1000 mg/kg) and negative control were 50.6 and 46.9, respectively. Whereas, the percentage difference BG level between AQ crude extract and AQ residue of AQ extract (1000 mg/kg) and normal control were 3.4 and 9.0, respectively.

## Discussion

This study investigated the *in vivo* antihypertensive effects of different extracts of *M. stenopetala* (Baker f.) Cufod. Leaves in D-Fructose (66% w/v *ad libitum*) induced hypertensive male Wistar rats. In addition, the effects on lipid profile and phytochemical screening of different extracts of *M. stenopetala* (Baker f.) Cufod. Leaves were done. This is the first indepth study to investigate the effect of different extracts of *M. stenopetala* (Baker f.) Cufod. Leaves on BP.

### Phytochemical screening

Phytochemicals are non-nutritive plant chemicals which may have some disease preventive or treatment properties. The four solvent extracts (AQ crude, 70% EtOH crude, EtAc partition, and AQ partition residue of AQ crude) of the fresh *M. stenopetala* (Baker f.) Cufod. Leaves were screened for the presence of different phytochemicals.

The qualitative phytochemical screening of AQ extract showed the presence of all tested secondary metabolites. This finding is in agreement with the study done on AQ extract of *M. oleifera* (Brindha et al., 2014). And 70% EtOH extract showed presence of all tested phytochemicals except saponins. This finding is inline with the study done on EtOH extract of *M. oleifera* (Onyekaba et al., 2013). Tannin and phytosterol were present in all tested extracts. Saponin was present in all tested extracts but not in 70% EtOH crude extract. Only crude extracts showed a positive test result for alkaloids and glycosides. One of the previous studies showed the presence of alkaloids, tannins, and glycosides but no saponins and anthraquinones in EtOH extract of *M. oleifera* (Denen et al., 2014). Another study showed the presence of all tested metabolites (tannin, alkaloid, saponin, and phenol) in both EtOH and EtAc crude extract of *M. oleifera* (Ojiako, 2014). On the otherhand, another study showed the presence of all tested metabolites (flavonoid, anthraquinone, alkaloid, saponin, terpenoid, glycoside, and tannin) in both EtOH and AQ crude extract of *M. oleifera* (Nweze and Felix, 2014). The present study indicated that the fresh leaves extracts of *M. stenopetala* (Baker f.) Cufod. contain different classes of secondary metabolites. The yield obtained for secondary metabolites of *M. stenopetala* (Baker f.) Cufod. leaves in the present study was recorded to be highest in the case of AQ crude extract followed by 70% EtOH crude, AQ residue, and EtAc fraction of AQ crude extract in succession. The presence of these phytochemicals gave a great potential for extracts of *M. stenopetala* leaves in producing vasodilatory effect that signifies the potential of the plant as a source of therapeutic agent.

### In *vivo* antihypertensive and antihyperlipidemic activity

#### Effect on blood pressure

The negative control rats which received 66% w/v D-Fructose served as hypertensive model with an average increase in SBP (50 mmHg), DBP (25 mmHg), and MAP (33 mmHg) from basal BP. Whereas, positive control which were given captopril (20 mg/kg/day) with 66% w/v D-Fructose *ad libitum* showed the average change in SBP (7 mmHg), DBP (−1 mmHg), and MAP (2 mmHg) were considered as normotensive after 15 days of study period.

Daily oral administration of the crude extracts of *M. stenopetala* (Baker f.) Cufod. significantly prevented the increase in SBP, MAP, and DBP in 66% w/v D-Fructose *ad libitum* consuming rats in a dose dependent manner. The highest daily oral dose of AQ crude extract (1000 mg/kg) significantly prevented the increase in SBP, MAP and DBP comparable to positive and normal control, groups that received captopril (20 mg/kg/day) and distilled water (*ad libitum*), respectively. The highest dose of 70% EtOH crude extract also produced a significant decrease in SBP, MAP, and DBP. Whereas, the EtAc fraction and AQ residue of AQ crude extract did not show significant decrease in SBP, MAP, and DBP, rather there was significant rise in SBP, MAP, and DBP comparable to that in negative control, groups that received only 66% w/v D-Fructose *ad libitum.* The AQ and 70% EtOH crude extract produced the highest dose dependent antihypertensive effect. This effect may be attributed to the presence of alkaloids and glycosides in crude extracts. This finding is in agreement with the study done on alkaloids of *M. oleifera* leaves on isolated frog heart (negative inotropic and chronotropic effect; Dangi et al., 2002), on glycosides isolates of *M. oleifera* leaves (Faizi et al., 1995).

#### Effect on total cholesterol, glucose, and triglyceride plasma levels

In this study, it was observed that the extracts decreased TC, BG, and TG plasma levels in a dose dependent manner indicating that they could prevent atherosclerosis. The level of TG however, increased with the extracts. This finding is in line with those of the previous studies carried out on antidiabetic and antihyperglycemic activity of different solvent extracts of *M. stenopetala* (Baker f.) Cufod. leaves using various models: 70% EtOH and its fractions in alloxan induced diabetic mice (Sileshi et al., 2014), n-butanol fraction of 70% EtOH in alloxan induced diabetic mice (Toma et al., 2012) and AQ, 70% EtOH and n-butanol fractions in STZ induced diabetic rats (Toma et al., 2015). The finding is also in agreement with those of the previous studies done on antidiabetic and antihyperlipidemic of different solvent extracts of *M. oleifera* leaves using various models: AQ extract in STZ and fructose induced rats (Divi et al., 2012), EtOH extract in STZ induced diabetic rats (Chinedu et al., 2014), EtOH extract in hypercholesterolemic rats (Denen et al., 2014), AQ extract in diabetic patients (Brindha et al., 2014), and 98% EtOH extract in alloxan induced rats (Aja et al., 2015).

In the present study, AQ crude and residue extract of *M. stenopetala* (Baker f.) Cufod. showed the suppression in BG level increment in a dose dependent manner. The highest suppression was observed at 1000 mg/kg which is comparable with normal rats. The TC, BG and TG plasma level suppression effect was high in groups that received AQ crude followed by 70% EtOH crude extract. The AQ crude extract (1000 mg/kg) has suppressed the TC, BG, and TC level by 87.2, 96.6, and 37.2% compared to normal control. The crude extracts showed dose dependent suppression in TC, BG, and TG level increment and the highest effect was observed at the maximum tested dose (1000 mg/kg/day).

## Conclusion

This study demonstrated the *in vivo* antihypertensive and antihyperlipidemic activity of the AQ and 70% EtOH crude extracts of *M. stenopetala* (Baker f.) Cufod. leaves in 66% w/v D-Fructose induced hypertensive male Wistar rats. Further studies, however, need to be done to confirm this using different model. Moreover, in-depth investigations are required in order to isolate and identify the phytoconstituents that are responsible for the plants antihypertensive and antihyperlipidemic activity as no prior studies have been undertaken in this regard.

## Author contributions

BG: Title selection, proposal writing and research design, laboratory experimentation (plant material preparation and extraction, phytochemical screening and *in vivo* antihypertensive and antihyperlipidemic) and result generation, data analysis and interpretation, manuscript writing, and submission. EM: Advising and edition in proposal and research design, result interpretation, and manuscript writing. AD: Advising and edition in proposal and research design, result interpretation, and manuscript writing. AT: Data feeding, analysis, and interpretation.

### Conflict of interest statement

The authors declare that the research was conducted in the absence of any commercial or financial relationships that could be construed as a potential conflict of interest.

## Abbreviations

CVD: Cardiovascular Disease
EPHI: Ethiopian Public Health Institute
EtOH: Ethanol
AQ: Aqueous
EtAc: Ethyl acetate
BP: Blood pressure
BP_0_: Basal blood pressure
SBP: Systolic blood pressure
MAP: Mean arterial blood pressure
DBP: Diastolic blood pressure
TC: Total cholesterol
BG: Blood glucose
TG: Triglyceride
D_5_: Fifth day
D_10_: Tenth day
D_15_: Fifteenth day
STZ: Streptozocin
ACEIs: Angiotensin Converting Enzyme Inhibitors
ARBs: Angiotensin II Receptor Blockers
CCBs: Calcium Channel Blockers.
